# Radius neck-to-humerus trochlea transposition elbow reconstruction after proximal ulnar metastatic tumor resection: case and literature review

**DOI:** 10.1186/2047-783X-17-23

**Published:** 2012-07-16

**Authors:** FeiYan Chen, Jun Xia, YiBing Wei, SiQun Wang, JianGuo Wu, GangYong Huang, Jie Chen, JingSheng Shi

**Affiliations:** 1 Department of Orthopedics, Huashan Hospital, Fudan University, Middle Urumqi Road No. 12, Shanghai, 200040, China

**Keywords:** Proximal ulna, Metastatic tumor, Reconstructive procedures, Elbow reconstruction, Ulnar reconstruction

## Abstract

Wide *en bloc* excision of proximal ulna sections is used to treat traumatic and pathological fractures of the ulna, though poor standardization of clinical treatment often results in long-term failure of such reconstructed biomechanical structures. In order to provide insight into effective ulnar reconstructive treatments, the case of an 80-year-old Chinese Han male presenting with pathological fracture caused by a proximal ulnar metastatic tumor concurrent with metastatic renal cancer complicated by occurrence in the brain and lungs is reported and contrasted with alternative treatment techniques. Wide resectioning of the proximal ulna and reconstruction with local radius neck-to-humerus trochlea transposition resulted in preservation of functionality, sensitivity, and biomechanical integrity after postsurgical immobilization, 6 weeks of passive- and active-assisted flexion, and extension with a hinged brace. The resultant Musculoskeletal Tumor Society rating score was 25 of 30 (83 %). Full sensitivity and mobility of the left hand and elbow (10° to 90° with minimally impaired supination and pronation) was restored with minimal discomfort. No evidence of local recurrence or other pathological complications were observed within a 1-year follow-up period. Efficient reconstruction of osseous and capsuloligamentous structures in the elbow is often accomplished by allografts, prosthesis, and soft tissue reconstruction, though wide variations in risk and prognosis associated with these techniques has resulted in disagreements regarding the most effective standards for clinical treatment. Current findings suggest that radius neck-to-humerus trochlea transposition offers a superior range of elbow movement and fewer complications than similar allograft and prosthetic techniques for patients with multiple metastatic cancers.

## Background

Reconstruction processes addressing the defects produced by wide excision of portions of the proximal ulna are difficult to treat because of the complex biomechanical interactions surrounding the hinge joint of the elbow. The function of the elbow relies on a complex combination of restraints achieved by the dynamic muscles of the elbow and the static bony and capsuloligamentous structures to which they are attached. Under normal conditions, valgus stress ranges from 31 % in extension to 33 % in 90° flexion, with the radial head acting as a secondary stabilizer, minimizing valgus instability [1]. Removal of significant portions of the ulna can reduce the stabilization provided by the radial head, resulting in symptomatically impaired mechanical function of the elbow. Subsequent to reconstructive surgery, improper healing may also represent significant impairments that are not immediately evident, but instead manifest as symptomatic sensitivity or mobility limitations months or years after the surgical procedure.

In order to successfully reconstruct the biomechanical interactions between osseous and capsuloligamentous structures required for normal elbow function, structural vascularized bone grafts with internal fixation, elbow arthrodesis, and custom or modular prosthetic elbow arthroplasty are often conducted in clinical settings [2,3]. Bone grafts induce restoration of natural osteoconduction, osteoinduction, and osteogenic cells, particularly when composite grafting is applied [4]. Arthrodesis, while commonly selected for the treatment of traumatic elbow damage, often results in improper healing, necessitating extensive rehabilitation before mobility is restored [5]. Prosthetic total elbow arthroplasty (TEA) often depends highly on the varus-valgus interplay between the humeral and ulnar components, and is rarely used to treat cases where the ulnar head is impaired [6]. The appropriate treatment choice varies highly among different cases, often related to the patient’s condition, pathology, previous mobility, pain level, and unique mobility requirements. Each of these techniques represents a unique set of risks for the development of complications, such as infection, prosthetic loosening, or fracture. Furthermore, these complications exhibit the tendency to occur immediately after the surgical procedure or much later in the patient’s life, suggesting that appropriate treatment choice may also vary by the patient’s projected lifespan, normal stress or activity levels, and overall mobility requirements. While many previous studies have examined the immediate benefits of these surgical techniques, long-term assessment of reconstructive surgeries is rarely reported despite the critical role of long-term outcomes in determination of appropriate treatment methodology.

In order to assess the 1-year effectiveness of radius neck-to-humerus trochlea transposition, the rare case of an 80-year-old Chinese Han male undergoing the procedure after wide excision of an advanced metastatic ulnar tumor is examined in detail, providing a powerful precedent for the potential long-term success of this procedure in stabilizing the elbow with minimal impact on the sensitivity and minimal mobility impairment. Few cases have reviewed both the immediate postsurgical and long-term outcomes of this technique [7,8], suggesting the need for formal evaluation of such cases in order to improve clinical treatments choices and overall patient outcomes.

## Case presentation

### Patient information and initial diagnosis

**Figure 1:**
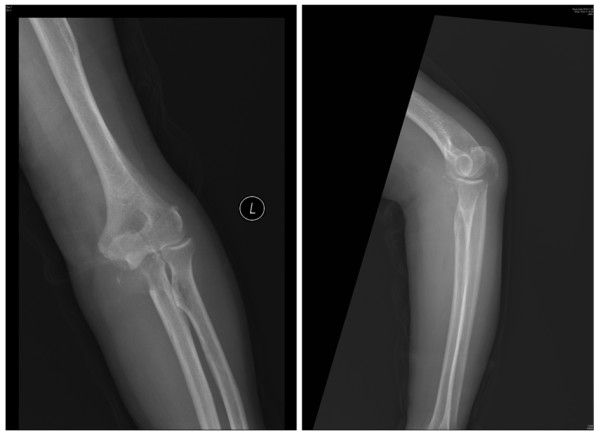
**Preoperative radiographic and computer tomographic (CT) images clearly indicate previously diagnosed and current lung metastatic tissues**.

An 80-year-old Chinese Han male with a history of recurrent renal cancer originating approximately 10 years prior was admitted to the present facility presenting a self-reported severe left forearm pain present for approximately 2 weeks. The patient reported treatment for recurrent renal cancer within the past 1–3 years, resulting in no apparent impairment to mobility or joint function. Metastasis of cancer tissue had previously been extensively observed in the patient, including brain metastasis treated with Gamma Knife procedures three times over the previous 7 years, as well as lung metastasis (Figure 1) first observed 5 years prior to the appearance of the currently described ulnar metastasis. Additionally, the patient had been previously advised to initiate chemotherapeutic treatment for lung and brain metastasis, but refused treatment based on the impact on his quality of life and independent living status. The patient was thus able to remain in independent living conditions with no in-home support or care prior to admission.

Following admission, the patient exhibited no abnormal vital signs. Specifically, no indication was found of hypertension, fever, dyspnea, or other abnormalities. Radiographic imaging revealed a fracture in the left proximal ulna. Coupled with the patient’s previous history of metastatic movement, these findings suggested an initial diagnosis of metastatic bone tumor and resultant pathologic fracture, a diagnosis that was confirmed prior to the procedure by routine biopsy of the affected area. Informed consent was obtained from the patient and the patient’s family for all procedures included in the current case study.

### Surgical treatment

**Figure 2:**
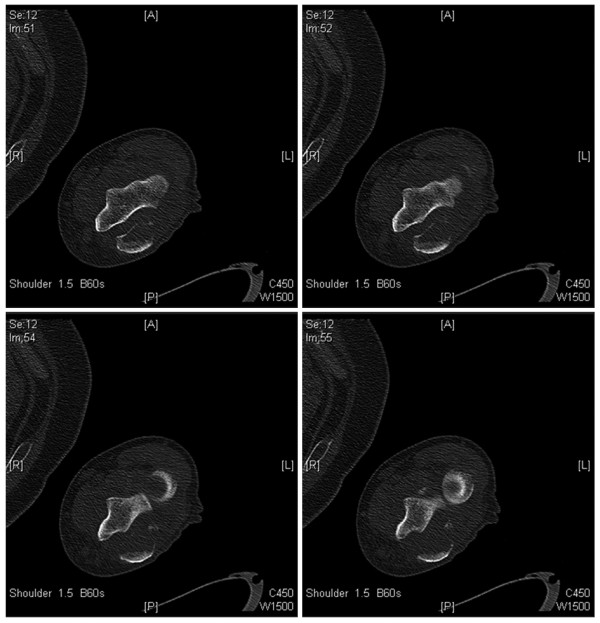
Preoperative radiograph of the left proximal ulna and pathologic fracture of the olecranon.

**Figure 3:**
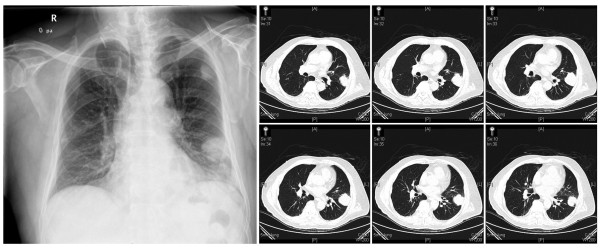
**Preoperative CT scan of the left elbow revealing signs of metastatic tumor along with destruction and pathologic fracture of the olecranon**.

Amputation of the affected limb was initially considered because of the extent and size of the lesion, generally considered indicative of poor reconstruction procedure outcomes resulting in limited mobility (Figures 2, 3). The patient was advised that limb-sparing treatment was likely to increase the risk of recurrent metastasis and would likely achieve poor reconstruction outcomes resulting in only partial restoration of mobility. Upon advisement, the patient expressed an explicit preference for a limb-sparing option, resulting in a treatment designed to provide localized disease control without amputation, offering maximum preservation of the forearm and elbow functionality. This treatment, however, is not generally recommended for limiting the risk of metastasis in recurrent patients with or without a history of metastatic growths.

The initial preoperative reconstruction plan recommended full elbow replacement with a custom prosthesis coupled with radius neck-to-humerus trochlea transposition. The patient and patient’s family opted to reject the prosthesis but expressed a desire to move forward with radius neck-to-humerus trochlea transposition. Due to the extremely wide tissue deficit expected after full removal of metastatic osseous tissues and surrounding materials, soft tissue coverage with a myocutaneous free flap was added to the surgical plan in lieu of a prosthetic graft.

To ensure optimal visibility during the operative procedure, a posterior approach was applied. The initial incision was drawn along the ulnar shaft, inclusive of the biopsy site. Identification of ulnar nerve was conducted visually, and the structure was subsequently exposed within the olecranon groove. The nervous tissue was carefully protected from surgical damage in order to maintain sensitivity in the forearm. Furthermore, the adjacent portions of the median and radial nerves were identified and similarly conserved in order to prevent postsurgical sensory damage.

During surgical removal of the tumor, wide *en bloc* excision was necessary for full removal of tumor tissues, resulting in the sacrifice of significant portions of the muscular elements attached to the tumor process. Specifically, large sections of the extensor muscle, supinator, and flexor pronator group adjacent to tumor tissues were excised, and the entire proximal half of the ulna was involved in the procedure. The margin of resection was 2 cm distal to the distal border of the tumor, as observed by T1-weighted magnetic resonance imaging, resulting in a relatively large resected area. Visual observation was used as the primary tool for resectioning of osseous and soft tissue regions, with negative margins determined according to the extent of observed malignancy. The procedure was completed in accordance with the guidelines previously provided by Enneking [2], wherein complete removal of malignant tissues were ensured by the application of margins a minimum of 5 cm distal to primary malignant bone tumors and 2 cm distal to metastatic tumors. Over the course of the operation, samples were collected from marginal tissues for frozen-section examination, resulting in definitive determination that margins were negative for metastatic tissue. A total length of 10 cm of the proximal ulna and surrounding soft tissue were resected.

**Figure 4:**
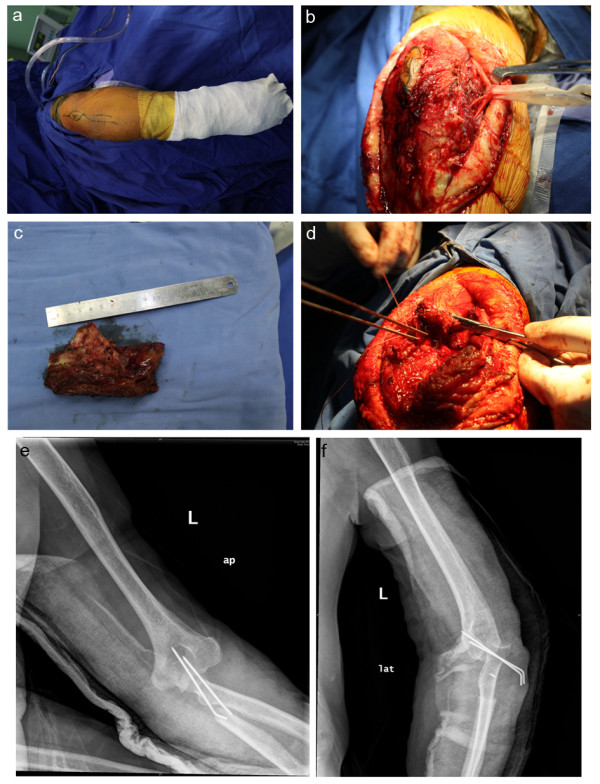
**(a) Posterior incision including the biopsy site.** (**b**) Exposure and preservation of the ulnar nerve. (**c**) Wide excision of the tumor segment. (**d**) Radius neck-to-humerus trochlear reconstruction; triceps tendon reconstructed to radius head using bone anchor and pins. (**e, f**) Postoperative radiograph showing cast and pins at 3 weeks.

The proximal radius was circumferentially mobilized, the radial-humeral joint was disarticulated, and the radial neck was then sufficiently mobilized to allow articulation with the humeral trochlea (Figure 4). The stability of the construct is considered to be highly dependent on the biceps tendon at its insertion point for normal movement, providing a mechanical purpose for soft restriction to posterior translation of the radius relative to the humeral trochlea. The triceps tendon was severed and reattached to the proximal radial head by soft tissue-to-bone repair, and the bond was further strengthened using a bone anchor.

### Tissue examination

Resultant tissue margins were examined as frozen sections, confirming the negative status for metastatic tissue.

### Surgical follow-up and outcomes

Notably, in cases where a small amount of the proximal olecranon can be spared in continuity with the tendon insertion, bone-to-bone repair is often successful. Reconstruction of the tendon insertion allows for subsequent extension against gravity and provides a restraint to translation of the radial neck as it articulates with the humeral trochlea. Additionally, the long-term stability of the reconstructed limb is highly dependent on the level of postoperative scar formation, which is often exacerbated by overstimulation in the weeks subsequent to surgery.

**Figure 5:**
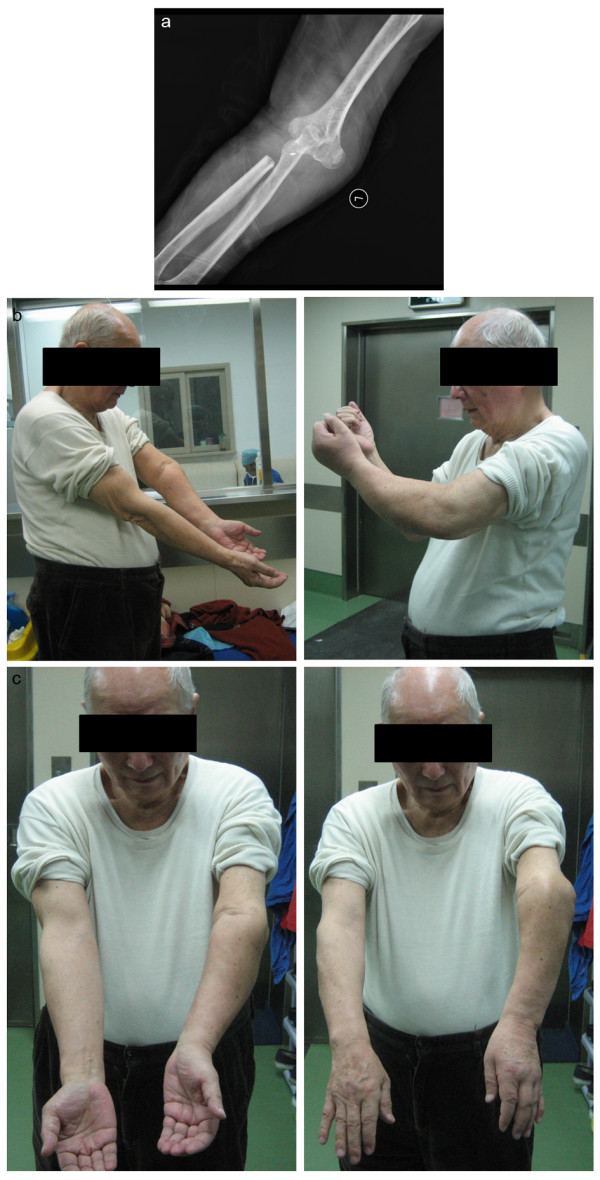
**Postoperative results for left elbow showing: (a) postoperative radiograph at 6-month follow-up, (b) extension and flexion, and (c) pronation and supination**.

In order to protect the reconstructed elbow joint, 3 weeks of full immobilization with a Kirschner pin and cast at 90° of flexion were ordered after surgery, followed by an additional 6 weeks of passive- and active-assisted flexion and extension in the sagittal plane with the added protection of a hinged brace. Good upper extremity function was observed after rehabilitation (Figure 5), though some tolerable discomfort was reported upon resuming normal daily activities.

The Musculoskeletal Tumor Society rating score for the patient after rehabilitation was 25 of 30 (83 %). The patient exhibited full sensitivity and use of the left hand, allowing the patient to successfully return to most normal activities of daily living with only moderate discomfort. Additionally, the range of the elbow movement was observed to be 10° to 90°, though supination and pronation were slightly restricted. No evidence of local recurrence or other pathological complications was observed after 1 year of follow-up. Due to the presence of stable renal cancer and concurrent lung and brain metastases, the patient remains at elevated risk for future metastatic occurrences.

## Discussion

The procedure of radial neck articulation with the trochlea was first described by Enneking in 1983 [2], although Dr. Cable Young was the first to perform the procedure in severe trauma cases. Bone tumors located at the proximal ulna are relatively rare, although reports of primary giant cell tumor, osteochondroma, and Ewing’s sarcoma of the proximal end of the ulna have appeared. Most osseous cancers, including those of the ulna, originate from the metastatic movement tumors localized in other tissues. A total of 2.5 % of all cases of ulnar tumor resulting in pathological fracture or symptomatic pain result from solitary metastasis of malignant cells from highly vascular renal cell carcinomas. Similar to the current case study, a single study reported application of a similar procedure for reconstruction after the wide resection of the proximal ulna to treat chondrosarcoma [8], resulting in highly positive outcomes in which the patient achieved a 35-135° range of motion at the elbow several weeks after the surgery and no chronic pain. Flexion and extension forces, however, were approximated at half of the normal strength observed in the contralateral limb. Both the previous case findings and the outcomes of the current case suggest that successful implementation of radius neck-to-humerus trochlea transposition for the reconstruction of the proximal ulna may improve prognosis compared with alternative techniques.

Alternatively, treatment with artificial elbow arthroplasty (AEA) has been widely reported, though numerous other techniques have also been employed for treatment of traumatic or pathological removal of large sections of osseous ulnar tissue [9,10]. Guo et al. [10] reported 19 cases of peri-elbow tumor resection and reconstruction using total elbow replacement (TER) prostheses. Evaluation by the Mayo Elbow Performance Score revealed that pain scores decreased from 3.6 to 2.0, and the mean arc of the elbow improved from 30° to 80°. An excellent or good result occurred in 14/19 of patients (77.8 %), and a poor result occurred in 4/19 patients (22.2 %). Common complications of total elbow replacement include infection, stem loosening, and peri-prosthesis fracture. Unfortunately, many of these effects may occur during the immediate postsurgical period or many months or years after the surgery, making the true long-term effectiveness of this technique difficult to evaluate. Revision is often necessary when these complications occur, which may result in additional scarring and the application of successive reconstructive techniques, often further impairing mobility and sensitivity. Specifically, elbow allografts offer additional control over scarring and reduced chances of rejection [9,11]. Because of the immediate benefits of reduced scaring, the potential for long-term failure and pathological complications is often overlooked in the choice to treat patients using arthroplastic techniques.

Vascularized fibular grafting is another viable technique used for reconstruction after excision of large areas of osseous tissues. Gianoutsos [11] reported this treatment following the removal of a hard bloc of cancerous adamantinoma bone tissue located in the proximal ulna, with good outcomes. Furthermore, Kimura et al. [8] reported a case of Ewing’s sarcoma of the proximal ulna treated with hemiarthroplasty coupled with a vascularized fibula graft, resulting in no local recurrence and excellent function achieved by the 4-year follow-up. Though outcomes of this treatment are generally positive, fracture, infection, and gradual degeneration remain prominent issue with its widespread implementation. In order to provide a contrast of the current results and results of previous studies, treatment outcomes and complications have been assessed in Table 1. The table clearly indicates that treatment with radius neck-to-humerus trochlea transposition is associated with greater restoration of range of elbow movement and improved resistance to future fracture, deterioration, and infection compared with the use of prostheses or grafting techniques. Though these results indicate an initial benefit for choosing radius neck-to-humerus trochlea transposition treatment, further studies conducted using much larger cohorts will be required to verify these findings before concrete clinical recommendations can be produced.

**Table 1:** **Treatment techniques and postoperative outcomes for wide*en bloc*excision of portions of the proximal ulna**

**Author**	**No. of patients**	**Treatment**	**Outcome**	**Common complications**
Current study	1	Radius neck-to-humerus trochlea transposition	Elbow movement of 10° to 90°, though supination and pronation were slightly restricted. Full upper limb sensitivity	Weakness of muscle strength and instability of the elbow joint
Anders Rydholm [8]	1	Radius neck-to-humerus trochlea transposition	Elbow movement of 35-135° range of motion, though flexion and extension forces were at half of the normal strength	Weakness of muscle strength and instability of the elbow joint
Guo et al. [3]	19	Total elbow replacement prostheses	Elbow movement improved from 30° to 80°. Excellent or good results occurred in 14/19 of patients (77.8 %).	Infection, stem loosening, and peri-prosthesis fracture requiring revision for some complications.
Gianoutsos [11]	1	Vascularized fibular grafting	Good outcome	Fracture, infection, and gradual Charcot-like degeneration
Kimura et al. [12]	1	Hemiarthroplasty with a vascularized fibula graft	Good outcome with no local recurrence and excellent function achieved by the 4-year follow-up	Fracture, infection, and gradual Charcot-like degeneration

For each technique short-term outcomes are often reported as excellent or good without adequate follow-up. Due to the consistent mechanical stressors associated with daily activities of the elbow joint, long-term outcomes that are most critical to the determination of proper treatment methodology often remain poorly documented. In many cases where other reconstructive options are contraindicated, radius neck-to-humerus trochlea transposition can be considered a viable alternative for reconstruction following wide resectioning of the proximal ulna or severe elbow trauma. Not only does this method offer superior elbow mobility after rehabilitation, as indicated by the present case study and several of its predecessors, this limb-sparing operation may likely provide superior long-term stability and result in less interference with limb sensitivity.

Additionally, the purpose of surgical therapy in the current case was primarily palliation and improvement in the quality of life remaining for the patient, not prevention of metastasis. It is important to note that the current patient’s previous metastases may complicate long-term follow-up, requiring additional studies to assess the effectiveness of this treatment for elbow reconstruction in patients that will resume periods of sustained or intensive elbow use.

## Conclusion

Although published data regarding neck-to-trochlea transposition are severely limited, resulting in a lack of clinical standards for treatment, the results of the current case study suggest that this option for reconstruction after wide excision of the proximal ulna results in the maximum restoration of elbow motion in the sagittal plane while avoiding many common complications associated with prosthetic or allograft reconstruction. Neck-to-trochlea transposition provides a viable clinical alternative that may be suitable for use in many clinical cases, suggesting that the technique should be carefully considered in cases of wide *en bloc* reconstructive surgery of the proximal ulna.

## Consent

Written informed consent was obtained from the patient(s) for publication of this manuscript and accompanying images. A copy of the written consent has been made available for review by the Editor-in-Chief of this journal.

## Competing interests

The authors declare that they have no competing interests.

## Authors’ contributions

Xia Jun and FeiYan Chen designed the surgical procedure. Xia Jun, YiBing Wei, and FeiYan Chen performed the operation. SiQun Wang, JianGu Wuo, and GangYong Huang participated the postoperative care and follow-up work. Jie Chen and JingSheng Shi performed the literature research and manuscript preparation. All authors contributed to the completion and approval of the final manuscript.
